# ALA and ALA hexyl ester in free and liposomal formulations for the photosensitisation of tumour organ cultures

**DOI:** 10.1038/sj.bjc.6600144

**Published:** 2002-03-04

**Authors:** A Casas, C Perotti, M Saccoliti, P Sacca, H Fukuda, AM del C Batlle

**Affiliations:** Centro de Investigaciones sobre Porfirinas y Porfirias (CIPYP) FCEyN (University of Buenos Aires and CONICET), Ciudad Universitaria, Pabellón II, 2do piso; (1428), Buenos Aires, Argentina; Servicio de Patología, Hospital Durand, Buenos Aires, Argentina

**Keywords:** aminolevulinic acid, photodynamic therapy, ALA, PDT, hexyl–ALA, ALA derivatives, liposomes

## Abstract

In spite of the wide range of tumours successfully treated with 5-aminolevulinic acid mediated photodynamic therapy, the fact that 5-aminolevulinic acid has low lipid solubility, limits its clinical application. More lipophilic 5-aminolevulinic acid prodrugs and the use of liposomal carriers are two approaches aimed at improving 5-aminolevulinic acid transmembrane access. In this study we used both 5-aminolevulinic acid and its hexyl ester in their free and encapsulated formulations to compare their corresponding endogenous synthesis of porphyrins. Employing murine tumour cultures, we found that neither the use of hexyl ester nor the entrappment of either 5-aminolevulinic acid or hexyl ester into liposomes increase the rate of tumour porphyrin synthesis. By light and electronic microscopy it was demonstrated that exposure of tumour explants to either free or liposomal 5-aminolevulinic acid and subsequent illumination induces the same type of subcellullar damage. Mitochondria, endoplasmic reticulum and plasma membrane are the structures mostly injured in the early steps of photodynamic treatment. In a later stage, cytoplasmic and nuclear disintegration are observed. By electronic microscopy the involvement of the endocytic pathway in the incorporation of liposomal 5-aminolevulinic acid into the cells was shown.

*British Journal of Cancer* (2002) **86**, 837–842. DOI: 10.1038/sj/bjc/6600144 www.bjcancer.com

© 2002 Cancer Research UK

## 

Photodynamic Therapy (PDT) of certain neoplasms has emerged as a promising form of cancer treatment involving the administration of a photosensitiser with subsequent application of light (Dougherty *et al*, 1978). The ensuing photodynamic reaction results in tumour destruction. PDT has shown promising results for superficial, small skin tumours and in internal hollow organs (Dougherty, 1993).

In a new modality of PDT, the endogenous production of the photosensitiser Protoporphyrin IX (PpIX) is induced by the prodrug 5-aminolevulinic acid (ALA) through the haem biosynthetic pathway. An advantage of the use of PpIX relative to other photosensitisers is the short half life of its photosensitising effects, which do not last longer than 48 h (Kennedy *et al*, 1990; Fukuda *et al*, 1993).

In spite of the wide range of tumours successfully treated with ALA–PDT, the fact that ALA is a zwitterion at physiological pH and therefore has low lipid solubility, limits its clinical application. More lipophilic ALA prodrugs are expected to cross cell membranes more easily than ALA. After entering the site of action, the prodrug is enzymatically converted to ALA, which in turn is converted into PpIX. Kloek and Beijersbergen van Henegouwen (1996); Kloek *et al* (1998) and Gaullier *et al* (1997) found that long chain ALA esters are taken up, hydrolysed to the free acid and converted into PpIX with better efficiency than ALA, leading to higher photosensitiser levels both *in vivo* and *in vitro*.

The use of the hexyl ester of ALA (He–ALA) proved to be particularly better than ALA for the photosensitisation of rat bladder cells (Cosserat-Gerardin *et al*, 2000) and murine mammary adenocarcinoma cells (Casas *et al*, 2001) and for the photodetection of human bladder cancer (Lange *et al*, 1999). However, the use of He–ALA for the treatment of skin cancer is still a matter of discussion, due to the fact that He–ALA diffuses more slowly across the stratum corneum than ALA (Van den Akker *et al*, 2000).

Liposomes have been used as drug carriers to increase efficacy of a variety of therapeutic agents, including antineoplastics, antibiotics and immunomodulators (Lasic, 1993). We demonstrated that intratumour and i.p. administration of liposome-entrapped ALA to tumour bearing mice, resulted in both increased porphyrin biosynthesis and higher tumour/normal tissue porphyrin ratio (Fukuda *et al*, 1992).

The aim of the present study was to measure porphyrin generation from ALA and its hexyl-ester derivative delivered in their free or liposomal formulations to murine mammary adenocarcinoma organ cultures. The existence of differential subcellular targets for PDT employing either free or liposomal ALA formulations was determined by means of electronic and light microscopy examination.

## MATERIALS AND METHODS

### Chemicals

ALA and phosphatidylcholine were purchased from Sigma Chemicals Co. (St. Louis, MO, USA). All other chemicals were of analytical grade.

### Hexyl–ALA synthesis

He–ALA was prepared according to the method of Takeya (1992) by reacting ALA with hexanol in the presence of thionyl chloride, yielding the product He–ALA as the hydrochloride salt (HCl).

### Preparation of liposomes

Large multilamellar liposomes (1 μ size) containing ALA and He–ALA were prepared according to Fukuda *et al* (1989) with the neutral phospholipid phosphatidylcholine. The resulting liposomal pellet was suspended in phosphate buffer saline (PBS). An encapsulation efficiency of 5% was determined measuring ALA and He–ALA concentration in the Triton-lysated pellet and in the supernatant by the Mauzerall and Granick (1956) method. The liposomes were used immediately after preparation.

### Animals

Male BALB/c mice 12 weeks old, weighing 20–25 g were used. They were provided with food (Purina 3, Molinos Río de la Plata) and water *ad libitum*. Innocula of the mammary adenocarcinoma M2, Instituto Roffo, Buenos Aires (Scolnik *et al*, 1984) were injected subcutaneously under the axilla. After 15 days of tumour growth, explants were excised from a unique tumour for each experiment. Animals were treated in accordance with guidelines established by the Animal Care and Use Committee of the Argentine Association of Specialists in Laboratory Animals (AADEALC), in full accord with the UK Guidelines for the Welfare of animals in Experimental Neoplasia (UKCCCR, 1988).

### Porphyrin determination

After ALA or He–ALA exposure, explants of 50 mg were homogenised in a 4 : 1 solution of ethyl acetate-glacial acetic acid mixture. Conditions and explant size were determined in previous work (Fukuda *et al*, 1989). The homogenates were centrifuged for 30 min at 3000 **g** and to the supernatants an equal volume of 5% HCl was added (Falk, 1964). Extraction with HCl was repeated until negative fluorescence in the organic layer. The aqueous fraction was used for the fluorimetric determination of porphyrins with excitation at 407 nm and emission at 604 nm. Calibration was with PPIX standard (Porphyrin Products, Palo Alto, USA) in 5% HCl.

### Tumour explant photodynamic treatment

Tumour tissue explants (4–5 mg) of non-necrotic, non-haemorrhagic tumour were floated in petri dishes in serum-free minimal essential Eagle's medium (MEM), supplemented with 2 mM L-glutamine and gentamycin (40 μg ml^−1^) and incubated at 37°C in presence of 0.6 mM ALA for 3 h. After irradiation with a fluence rate of 190 J cm^2^, medium was replaced by FBS-containing medium until fixation for microscopic examination. Appropriate controls of non-irradiated explants incubated with ALA, and irradiated and non-irradiated explants incubated in the absence of ALA were performed.

### Laser irradiations

Irradiations were performed employing a rhodamine dye laser (Model DL30, Oxford Lasers) pumped by a copper vapour laser (CU15A, Oxford Lasers) tuned to 630 nm. The light was focused into a 400-μm-diameter optical fibre coupled to a frontal light distributor (Model FD2, Medlight, Ecublens, Switzerland) to produce a treatment area of uniform intensity. The output power from the fibre was measured with a power meter (Model LM-100XL, Coherent, Auburn, CA, USA) before each illumination and adjusted to the desired light dose.

### Electron and light microscopy

Explants were fixed 6 h at 4°C in 3% glutaraldehyde in Millioing buffer (Sodium phosphate buffer 440 μOsm pH 7.3), post-fixed in 2% osmium tetroxide in 0.1 mM sodium cacodylate buffer pH 7.2, dehydrated in ethanol and embedded in epon 812 (epoxy resin). Thin sections (750–900 Å) were cut in a ultramicrothome Sorvall Portem-Blum MT2B model after staining with uranyl acetate 2% Reynolds lead citrate and then observed in a Zeiss EM109 transmission electron microscope. Specimen stained with toluidine blue were examined by light microscopy.

### Statistics

Results are presented as the mean±standard deviation of three duplicate experiments and comparisons between dose points were made with the two-tailed *t*-test. Values of *P*<0.05 were considered significant.

## RESULTS

### Porphyrin accumulation from free and liposomal ALA and He–ALA

Figure 1

**Figure 4:**
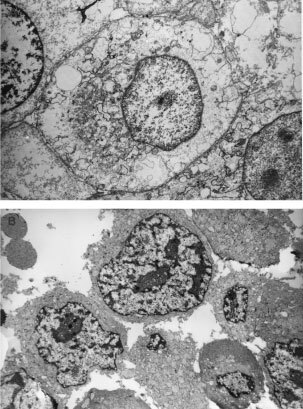
Transmission electron micrograph of control and ALA–PDT treated explants. (**A**) Explant incubated 3 h in presence of 0.6 mM free ALA, immediately irradiated with 190 J cm^2^, and fixed 4 h after. Mitochondrial and endoplasmic swelling can be observed. Loss of interstitial space due to general cell enlargement (×3000). (**B**) Control explant incubated in the same conditions in absence of ALA and non-irradiated (×3000).

**Figure 1:**
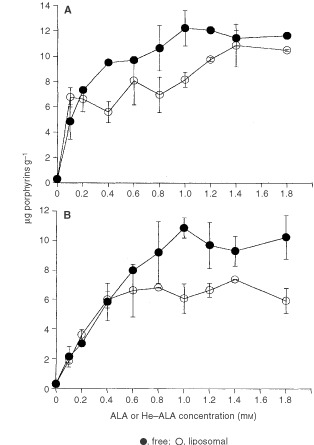
Porphyrin synthesis from free and liposomal ALA and He–ALA in tumour explants. Tumour explants were incubated 3 h in the presence of different concentrations of free or liposomal ALA (**A**) or He–ALA (**B**) in FBS-free medium. Tissue porphyrins were extracted and determined fluorimetrically. The values are expressed as μg porphyrins g^−1^ tissue.

depicts porphyrin synthesis in presence of different free or liposomal ALA and He–ALA concentrations. We can observe a saturation point at 1 mM free ALA (12.20±1.39 μg porphyrins g^−1^ tissue) and 1.4 mM liposomal ALA (10.83±1.64 μg g^−1^). Saturation points are also observed for free and liposomal He–ALA: 10.88±0.68 μg g^−1^ for 1 mM free He–ALA and 6.56± 0.14 μg g^−1^ for 0.4 mM liposomal He–ALA.

At low concentrations of both pro-photosensitisers (up to 0.2 mM ALA and 0.4 mM He-ALA) there are no significative differences in the amount of porphyrins generated, independently of the vehicle. However, at higher concentrations, values are impaired by the use of the liposomal formulations from 10 up to 40% depending on the concentration employed. This pattern can be seen in both ALA and He–ALA treated explants.

In Figure 2

**Figure 2:**
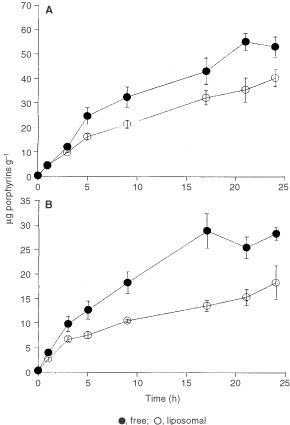
Porphyrin synthesis as a function of incubation time in the presence of free and liposomal ALA and He–ALA in tumour explants. Tumour explants were incubated during different time periods in the presence of free or liposomal ALA (**A**) or He–ALA (**B**) in FBS-free medium. Tissue porphyrins were extracted and determined fluorimetrically. The values are expressed as μg porphyrins g^−1^ tissue.

we can observe the kinetics of intracellular porphyrin accumulation. The cellular porphyrin content increases linearly during the 24 h-period employing both ALA and He–ALA formulations. When using the free compounds, a plateau is reached at nearly 20 h of incubation. Porphyrin biosynthesis is always higher employing ALA than He-ALA in both formulations and the porphyrin accumulation ratios between the compounds increase further from 5 h incubation onwards.

Porphyrins released to media are about 30–35% of the amount retained intracellularly for all the concentrations of ALA and He–ALA tested and for both formulations (data not shown).

### Histological studies

**Figure 3:**
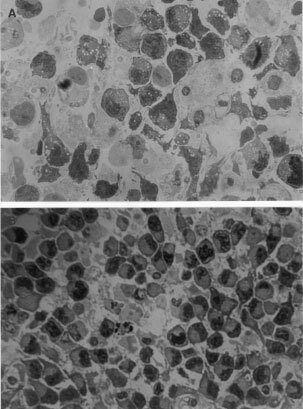
Light micrograph of free ALA–PDT treated explant and non-treated control. (**A**) Explant incubated 3 h in presence of 0.6 mM free ALA, immediately irradiated with 190 J cm^2^, and fixed 4 h after. Vacuo lization and vesiculation of the cytoplasm can be observed. Nuclei remain intact (×1000). (**B**) Control explant incubated in the same conditions in absence of ALA and non-irradiated (×630).

Four hours after either free or liposomal ALA–PDT treatment, some of the most common early alterations such as vacuolization and vesiculation of the cytoplasm were observed by light microscopy (Figure 3A

). Severe mitochondrial and endoplasmic swelling, membrane disruptions and invaginations, loss of interstitial space due to general cell enlargement can be observed in electronic micrographs (Figures 4

**Figure 5:**
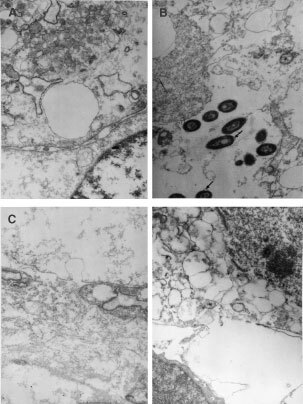
Transmission electron micrograph of a ALA–PDT treated explants. Explants were incubated 3 h in 0.6 mM ALA in free (**A**, **C** and **D**) or liposome-encapsulated (**D**), and immediately irradiated with 190 J cm^2^, and fixed 4 h after. (**A**) Details of mitochondrial and endo plasmic swelling (×7000). (**B**) Detail of ALA liposomes, surrounded by cell membrane (arrows) (×7000). (**C**) Membrane disruption (×7000). (**D**) Membrane invagination (×7000).

and 5

**Figure 6:**
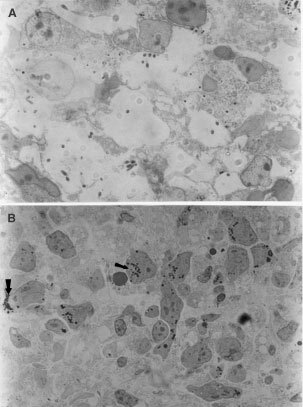
Light micrograph of liposomal ALA–PDT treated explant and non-treated control. (**A**) Explant incubated 3 h in presence of 0.6 mM liposomal ALA, immediately irradiated with 190 J cm^2^, and fixed 24 h after. Loss of cellularity can be observed, cell debris and free nuclei (×1000). (**B**) Control explant incubated with liposomal ALA and non-irradiated. Liposomes can be seen in the cytoplasm (small arrow), and some in the intracellular space just about to be either endocytosed or fused with the plasma membrane (large arrow) (×1000).

). Instead nucleous remained intact.

Employing the same light dose, 24 h after PDT, damage progressed and extensive tumour cell death injury, death and necrosis, cellular debris and nuclear remnants can be observed (Figure 6A). These features of early and late damage stages are identical in free and liposomal ALA–PDT treated explants.

Free ALA-treated and light-treated control explants, appeared normal and identical to non-ALA non-light explants illustrated in Figure 3B.

In explants treated with liposomal ALA and not irradiated, phospholipid vesicles can be seen in the cytoplasm and some in the intracellular space just about to be incorporated into the cell (Figure 6B). When these explants are illuminated, some free liposomes are still observed among cellular debris (Figure 6A). Details of liposomes surrounded by cell membrane are shown in an electronic micrograph (Figure 5B) and a huge lisosome containing a lot of liposomes is also observed (data not shown).

## DISCUSSION

Entrappment of either ALA or He–ALA into liposomes does not increase the rate of tumour porphyrin synthesis employing our tissue explant culture model. Moreover, liposomal exposure of the tumour cell line LM2 derived from this M2 mammary adenocarcinoma to either liposomal ALA or He–ALA produces less PpIX as compared to their free formulations (Casas *et al*, unpublished results). In contrast, *in vivo* administration of liposomal ALA produced an increased porphyrin accumulation in the tumour tissue (Fukuda *et al*, 1992). Differences between *in vivo* and *in vitro* results may be ascribed to the lack of a functioning vascular system and should be taken into account to assess the real performance of PDT treatment.

The use of He–ALA does not improve porphyrin synthesis either, which is instead significantly lower when compared with ALA in most cases. In partial agreement with these results, in previous work (Casas *et al*, 1999) we found employing rat skin explants that porphyrin synthesis from He–ALA only surpass synthesis from ALA at certain incubation times.

Employing the LM2 cell line (Casas *et al*, 2001) we had found that the maximum amount of porphyrins was synthesised from 0.6 mM ALA and that the same amount was produced by a concentration 60-fold lower of He–ALA. We had also shown that porphyrins formed from either ALA or ALA-esters equally sensitise the cells to photoinactivation. These surprisingly huge differences between results obtained with cell lines and parental tumours may be ascribed to a large number of factors: (a) diminished ability of He–ALA to cross vascular structures in the organ explants and then reach tumoural cells; (b) retention of He–ALA in the interstitial space and consequent limited availability to cell layers; (c) differential expression and activity of esterase in the cell line as compared to the parental tumour.

Despite these differences, alternative approaches can be designed in order to determine the efficacy of PDT that can better reflect an *in vivo* environment, such as controlling oxygen tension to mimic the low pO_2_ present in the tumour.

It has been shown that liposomes are endocytosed and/or fused with plasma membranes, thereby introducing their content into the cytoplasm (Margolis *et al*, 1982). The presence of huge lisosomes containing liposomes is an evidence at least of the participation of the endocytic pathway in the release of the entrapped ALA into the cell. Moreover, the presence of liposomes surrounded by cell membrane accounts for the endocytic pathway of liposome incorporation.

We do not know yet the localisation of either ALA or He–ALA within the liposome, but the hydrophobic nature of the ALA molecule would indicate that it should very likely be retained in the internal aqueous spaces between lipid layers in the core of the multilamellar liposomes. Because He–ALA is more lipophilic than ALA, it might be distributed between the aqueous and lipid phases of the liposomes; therefore their respective release from liposomes are expected to be different.

The permeability of liposomes to entrapped solutes increases when they interact with cells, plasma or serum, as well as with specific plasma proteins, especially lipoproteins. Lipids can exchange between liposomes and liporoteins and in some cases complete liposome breakdown can occur. In our case, the use of FBS-free medium is minimising this effect. In addition, liposomes composed of neutral phospholipids such as phosphatidylcholine, are more stable, and only 24% of their components are lost in 7 h (Hunt, 1982).

There were no differences between the subcellular damage induced by liposomal ALA–PDT when compared with free ALA–PDT, indicating that induced mechanisms of cellular death are the same. PpIX is generated in the mitochondria, re-distributed over the cytoplasm and into membranes with consequent damage of mitochondria and endoplasmic reticulum (ER). Direct mitochondrial damage has been addressed as the major target of ALA–PDT (Iinuma *et al*, 1994). The ER and other membranous structures might be damaged indirectly by reactive oxygen intermediates coming from the lipophilic sensitiser PpIX without direct targeting. The nucleous, however, remained unaffected in the early steps of cell damage induced by either free or liposomal ALA–PDT but later changes inducing necrotic cell death, include nuclear structure disruption.

Further studies are still needed to determine whether attempts to improve ALA–PDT treatment such as chemical modifications of the ALA molecule and ALA delivery within liposomal carriers will in fact be as useful as expected.
